# Gastrointestinal Histoplasmosis Mimicking Intestinal Tuberculosis and Crohn's Disease: Ileocecal Involvement in an Immunocompetent Host

**DOI:** 10.1002/ccr3.73555

**Published:** 2026-09-17

**Authors:** Rashid Shahriar Sazal, Sayma Samia Esha, Abdullah Al Faisal, Md. Abul Kalam Azad, Khaled Mahbub Murshed, Kazi Ali Aftab

**Affiliations:** ^1^ Department of Internal Medicine Bangladesh Medical University Dhaka Bangladesh

**Keywords:** Crohn's disease, diagnostic mimicry, disseminated histoplasmosis, fungal infection, gastrointestinal histoplasmosis, histopathology, Histoplasma capsulatum, ileocecal disease, immunocompetent host, intestinal tuberculosis

## Abstract

Gastrointestinal histoplasmosis is an under‐recognized manifestation of disseminated Histoplasma capsulatum infection. Its ileocecal predilection closely mimics intestinal tuberculosis (TB) and Crohn's disease, posing serious diagnostic challenges, particularly in TB‐endemic regions. A 26‐year‐old immunocompetent male presented with a 3‐month history of diffuse abdominal pain, hematochezia, and 16 kg weight loss. He had received 60 days of empirical four‐drug anti‐tubercular therapy (ATT) without improvement. Examination revealed cachexia, pallor, digital clubbing, pedal oedema, a hard palate ulcer, and small cervical lymphadenopathy. Investigations showed microcytic anemia, C‐reactive protein (CRP) 143.35 mg/L, hypoalbuminemia (2 g/dL), hyponatremia, and fecal calprotectin of 1849.47 μg/g. Magnetic resonance enterography (MRE) suggested multi‐segmental small bowel disease consistent with Crohn's disease. Sequential colonoscopies revealed ulcero‐nodular lesions involving the distal ileum, ileocecal valve, and caecum. Initial biopsies were inconclusive. Repeat deep tissue biopsy from the caecum and ileocecal valve identified intracellular narrow‐based budding yeast forms with eccentric nuclei, consistent with H. capsulatum. GeneXpert and Quantiferon‐TB gold plus were negative. Cervical lymph node fine‐needle aspiration cytology (FNAC) confirmed disseminated disease. HIV testing was negative. Itraconazole was initiated, followed by liposomal amphotericin B. On day 15, the patient developed catastrophic lower gastrointestinal hemorrhage, resulting in refractory hemodynamic collapse and death. Disseminated histoplasmosis should be considered in the differential diagnosis of ileocecal disease even in immunocompetent patients from TB‐endemic regions. Histopathological confirmation through deep tissue biopsy is critical prior to immunosuppressive therapy. Delayed diagnosis carries significant mortality risk.

## Key Clinical Message

Disseminated histoplasmosis may rarely present in immunocompetent individuals with ileocecal involvement mimicking intestinal tuberculosis or Crohn's disease. Elevated fecal calprotectin and radiologic findings are non‐specific. Failure of empirical ATT should prompt evaluation for fungal etiologies, and repeat deep tissue biopsy with fungal staining is crucial before initiating immunosuppressive therapy.

**Abbreviations**
ACTH: adrenocorticotropic hormone
ATT: anti‐tubercular therapy
CRP: C‐reactive protein
ELISA: Enzyme‐linked immunosorbent assay
FNAC: fine‐needle aspiration cytology
GMS: Gomori methenamine silver
HIV: Human immunodeficiency virus
IBD: inflammatory bowel disease
ICU: Intensive care unit
LDH: lactate dehydrogenase
MCH: mean corpuscular hemoglobin
MCV: mean corpuscular volume
MRE: magnetic resonance enterography
PAS: periodic acid–Schiff
TB: tuberculosis

## 1 Introduction

Histoplasma capsulatum is a thermally dimorphic fungus endemic to the Mississippi and Ohio River valleys of North America, with additional foci of endemicity across Central and South America, parts of Africa, and select Asian regions including the Indian subcontinent [1]. Infection is acquired via inhalation of microconidia from contaminated soil, particularly in environments associated with bat or bird guano, such as caves, poultry farms, and disturbed soil [2]. Pulmonary histoplasmosis is the predominant manifestation, but progressive disseminated histoplasmosis (PDH) occurs in a subset of patients, classically those with defective cellular immunity such as HIV/AIDS, organ transplant recipients, or individuals receiving immunosuppressive biologics.

Gastrointestinal histoplasmosis, observed in up to 70%–90% of autopsy series of PDH, is rarely diagnosed ante‐mortem [3]. Clinical presentations encompass abdominal pain, weight loss, diarrhea, and lower gastrointestinal bleeding, overlapping substantially with intestinal tuberculosis (TB) and Crohn's disease. Ileocecal involvement is particularly characteristic and may produce endoscopic findings including ulcero‐nodular lesions, strictures, and pseudopolyps that are indistinguishable from TB or IBD on routine endoscopy [4].

The occurrence of disseminated histoplasmosis in immunocompetent individuals, while recognized, remains diagnostically challenging, particularly in high‐TB‐burden regions where clinicians frequently operate within an anchoring bias framework favoring mycobacterial disease [5]. In such settings, failure to consider fungal etiologies can lead to harmful empirical ATT or, more critically, premature immunosuppressive therapy for presumed IBD, with potential for catastrophic outcomes. We report a fatal case of disseminated histoplasmosis with ileocecal involvement in an immunocompetent young male, initially misdiagnosed as intestinal TB and Crohn's disease, emphasizing the challenges of delayed diagnosis.

## 2 Case Presentation

### 2.1 History and Presentation

A 26‐year‐old unmarried male presented with a 3‐month history of progressive diffuse abdominal pain, hematochezia, anorexia, and 16 kg unintentional weight loss. Symptoms began as postprandial epigastric discomfort, worsened despite self‐administered acid suppression, and evolved to periumbilical pain. Prior colonoscopy at an outside facility was considered consistent with either intestinal TB or inflammatory bowel disease (IBD). Given regional TB endemicity and a strong family history of TB, empirical four‐drug fixed‐dose combination ATT was initiated. Despite documented compliance for 60 days, no clinical improvement occurred and ATT was discontinued.

On directed inquiry, the patient denied fever, night sweats, cough, arthralgias, or cutaneous manifestations. Travel history included recent cave exploration in hilly terrain, without bird exposure. There was no history of corticosteroid use, diabetes mellitus, hypertension, substance abuse, or high‐risk sexual behavior.

### 2.2 Physical Examination

On admission, the patient appeared cachectic and acutely unwell. General examination revealed marked pallor, bilateral digital clubbing, and bilateral pitting pedal oedema. Vital signs were within normal limits. A solitary, well‐defined ulcer was identified over the hard palate. Multiple small, firm, non‐tender cervical lymph nodes were palpable bilaterally, initially considered to be reactive. Abdominal examination revealed periumbilical tenderness without guarding, rigidity, or organomegaly. No abdominal masses were palpable. Cardiorespiratory and neurological examinations were unremarkable.

### 2.3 Investigations

Initial laboratory investigations (Day 1) revealed microcytic hypochromic anemia (hemoglobin 8.6 g/dL, MCV 69.5 fL, MCH 21.8 pg) and markedly elevated C‐reactive protein (CRP) at 143.35 mg/L. Hepatic and renal function tests were within normal limits. Biochemical profiling identified significant hypoalbuminemia (2 g/dL) and hyponatremia (serum sodium 127 mmol/L; chloride 89.7 mmol/L). Chest radiograph demonstrated mild bilateral pleural effusions. Lactate dehydrogenase was 281 U/L. Given persistent hyponatremia and the clinical context of possible adrenal involvement (Day 2), adrenal insufficiency was considered; however, morning serum cortisol (406.8 nmol/L) and ACTH (56.21 pg/mL) were both within reference ranges. Hepatitis B surface antigen and anti‐HCV antibodies were negative.

Magnetic resonance enterography (MRE) (Day 3) demonstrated multi‐segmental bowel wall thickening involving the distal jejunum and ileum, without stricture formation, and multiple sub‐centimeter mesenteric lymph nodes of probable reactive significance. The overall radiological impression favored Crohn's disease. Fecal calprotectin was markedly elevated at 1849.47 μg/g, and stool microscopy revealed 8–12 pus cells per high‐power field with mucus, further supporting an inflammatory intestinal process.

### 2.4 Endoscopic Evaluation and Histopathology

**FIGURE 1:**
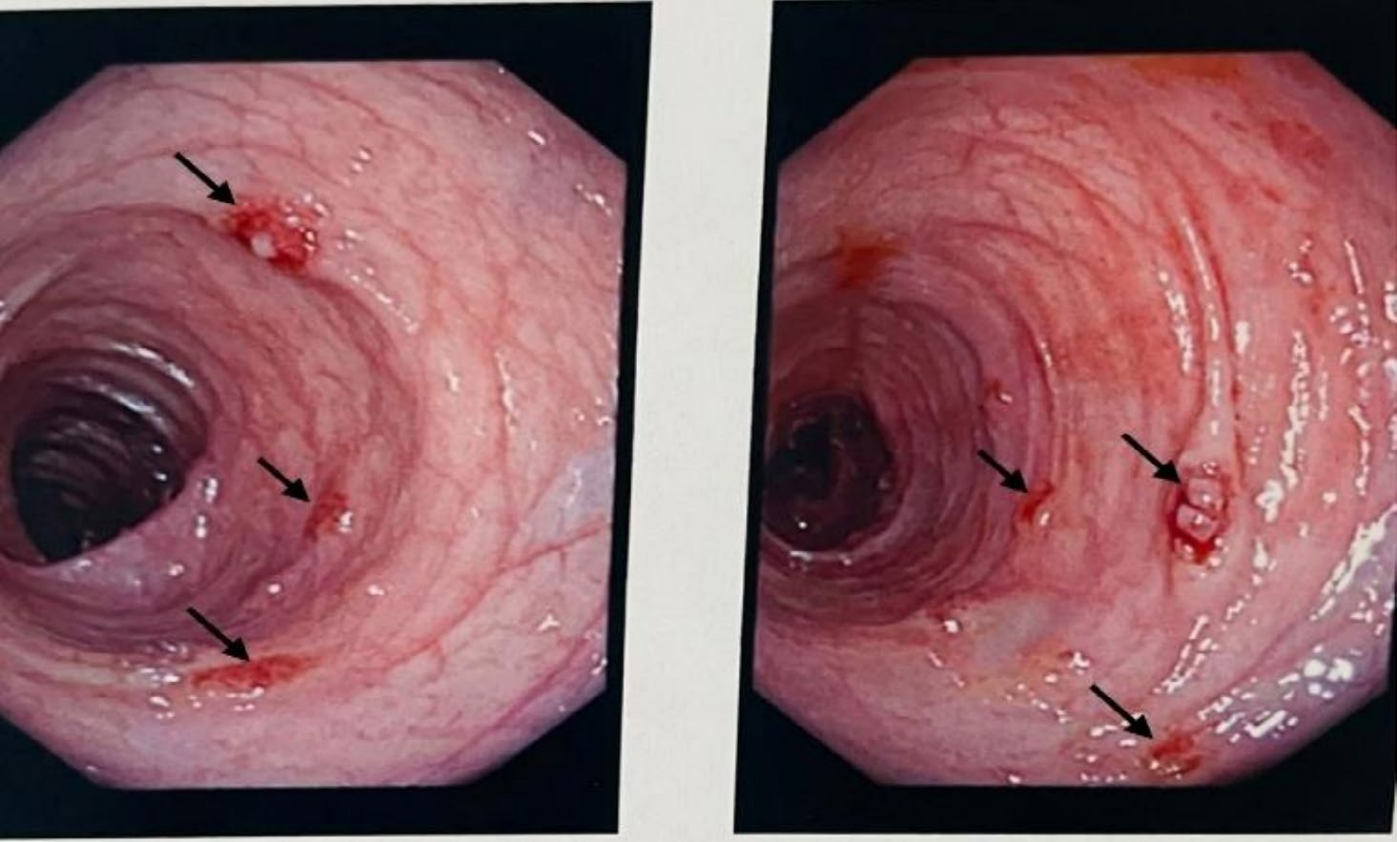
Black arrows showing ulcero‐nodular lesion with normal intervening mucosa in the terminal ileum, IC valve.

Upper gastrointestinal endoscopy (Day 5) identified a small benign‐appearing ulcer in the second part of the duodenum. Colonoscopy demonstrated an ulcero‐nodular lesion involving the caecum with multiple pseudopolyps distributed across the ascending, transverse, and descending colon (Figure 1). The differential diagnosis remained intestinal TB versus Crohn's disease. GeneXpert MTB/RIF assay performed on tissue was negative for *Mycobacterium tuberculosis* . Histopathological examination revealed granulation tissue with acute and chronic inflammatory infiltration (Figure S1), without definitive granuloma formation or evidence of malignancy, and was therefore diagnostically inconclusive.

**FIGURE 2:**
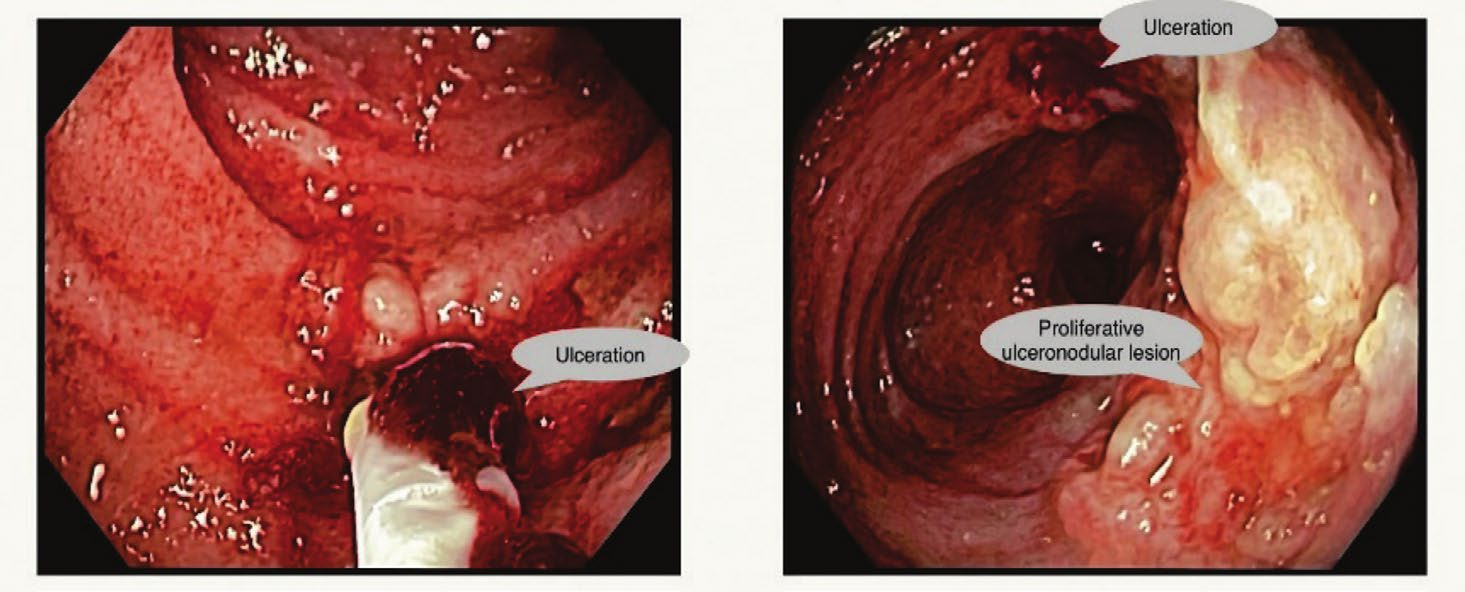
Repeat Colonoscopy showing ulcero‐nodular lesion with normal intervening mucosa in the terminal ileum, IC valve.

In view of ongoing clinical deterioration, a second colonoscopy was performed on Day 9. This confirmed ulcero‐nodular lesions with skip‐pattern involvement of the distal ileum, ileocecal valve, and caecum, alongside multiple small pseudopolyps at the aforementioned sites and throughout the colon (Figure 2). Repeat tissue sampling was obtained. Given persistent diagnostic uncertainty despite two endoscopic evaluations, fine‐needle aspiration cytology (FNAC) of the right cervical lymph node was performed on Day 11 for tissue diagnosis from an alternative accessible site.

**FIGURE 3:**
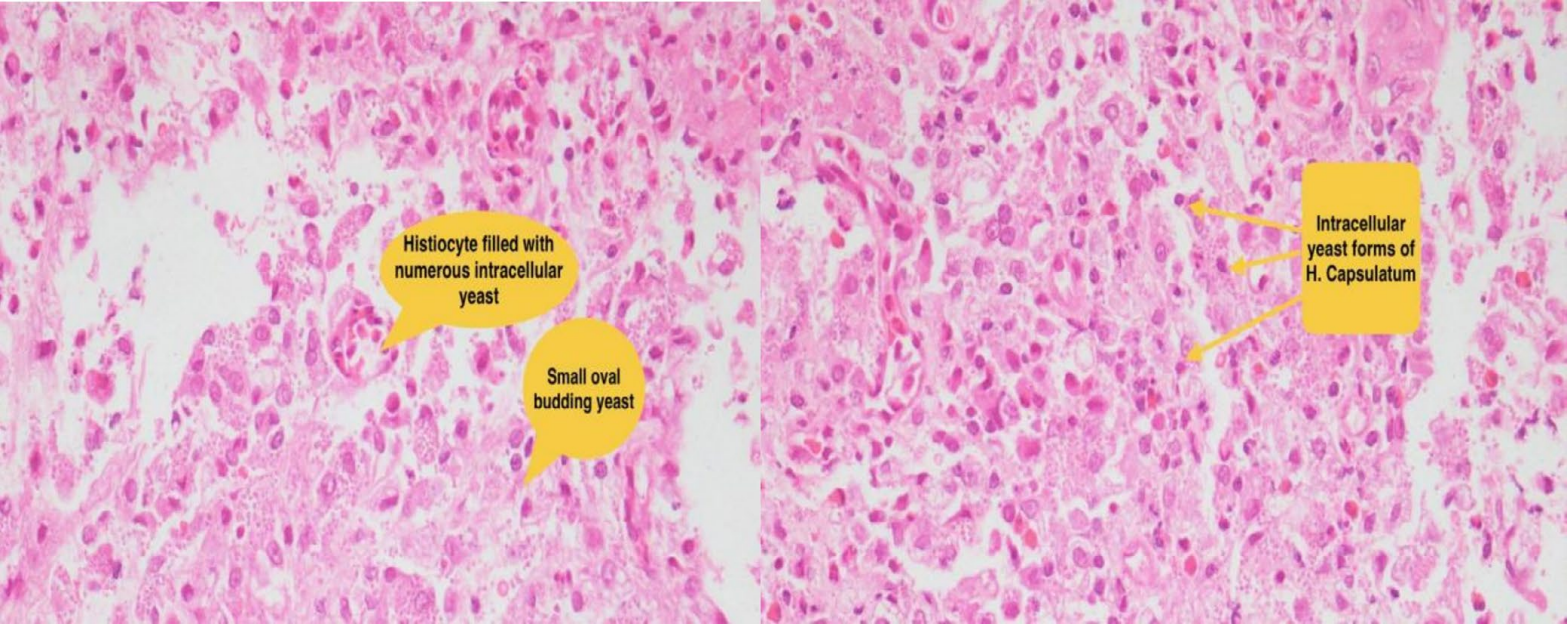
Sections show colonic mucosa with dense mixed inflammatory infiltrates and increased eosinophils in the lamina propria. Numerous small oval narrow‐based budding yeast forms are seen within and outside histiocytes, consistent with histoplasmosis.

Histopathological examination of cecal and ileocecal valve biopsies (Day 12) established the diagnosis. Microscopy demonstrated dense lamina propria infiltration by acute and chronic inflammatory cells with conspicuous eosinophilia. Crucially, multiple encapsulated, uniform, small, oval yeast forms exhibiting narrow‐based budding and eccentric nuclei were identified both within and outside histiocytes—morphological features diagnostic of Histoplasma capsulatum (Figure 3). GeneXpert MTB/RIF assay performed on tissue was again negative for *Mycobacterium tuberculosis* . Quantiferon‐TB gold plus ELISA was also non‐reactive. Oral itraconazole 200 mg twice daily was initiated immediately upon diagnosis. The following day (Day 13), cytopathological evaluation of the cervical lymph node aspirate demonstrated lymphocytes, histiocytes, and clusters of epithelioid cells on a hemorrhagic background, with numerous intracellular and extracellular Histoplasma organisms, further confirming disseminated disease (Figure S2).

### 2.5 Treatment and Outcome

Systemic treatment was escalated to liposomal amphotericin B (3 mg/kg/day) commencing Day 14. HIV serology, performed simultaneously, returned negative, establishing the diagnosis of disseminated histoplasmosis in an immunocompetent host. Following administration of two doses of liposomal amphotericin B, the patient developed a catastrophic episode of massive lower gastrointestinal hemorrhage on Day 15, accompanied by acute hemodynamic instability (blood pressure unrecordable, oxygen saturation 85% on 15 L/min supplemental oxygen). Despite immediate resuscitative efforts including blood transfusion, intravenous fluid resuscitation, and vasopressor support, the patient was transferred to the intensive care unit and succumbed approximately 6 h later due to refractory cardiorespiratory failure.

#### 2.5.1 Timeline

Table 1 summarizes the events, investigations, and diagnostic significance.

**TABLE 1:** Clinical timeline of events.

Day	Clinical event	Investigation/Intervention	Diagnostic significance
3 months prior	Abdominal pain, hematochezia, 16 kg weight loss; empirical ATT (60 days, no improvement)	Outside colonoscopy: TB vs. IBD; four‐drug ATT started	ATT failure raised suspicion for alternative diagnosis
Day 1	Admission; cachexia, pallor, clubbing, pedal oedema, oral ulcer, cervical lymphadenopathy	CBC, CRP, LFT, RFT, electrolytes, chest X‐ray	Microcytic anemia; CRP 143.35 mg/L; albumin 2 g/dL; hyponatremia; bilateral pleural effusions
Day 2	Persistent hyponatremia; adrenal involvement considered	Serum cortisol, ACTH, hepatitis B/C serology, LDH	Adrenal insufficiency excluded; viral hepatitis negative
Day 3	Clinical deterioration; Crohn's disease favored radiologically	MRE, fecal calprotectin (1849.47 μg/g), stool microscopy	Multi‐segmental bowel wall thickening; elevated calprotectin; immunosuppression deferred
Day 5	Upper GI and lower GI endoscopy	Duodenal ulcer; cecal ulcero‐nodular lesion; colonic pseudopolyps; biopsies obtained	Inconclusive histopathology: granulation tissue, no granulomata
Day 9	Ongoing deterioration; repeat colonoscopy	Ulcero‐nodular lesions: distal ileum, ileocecal valve, caecum; colonic pseudopolyps; deeper biopsies	TB vs. Crohn's differential persisted; deeper tissue sampling obtained
Day 11	Persistent diagnostic uncertainty	FNAC of right cervical lymph node	Extra‐gastrointestinal tissue sought to break diagnostic impasse
Day 12	Diagnosis established from cecal/ileocecal valve biopsy	Histopathology: narrow‐based budding yeast, eccentric nuclei; GeneXpert negative; Quantiferon‐TB gold plus negative; itraconazole started	H. capsulatum confirmed; mycobacterial disease excluded molecularly
Day 13	Lymph node FNAC result available	Intracellular and extracellular Histoplasma organisms; granulomatous inflammation	Disseminated histoplasmosis confirmed; liposomal amphotericin B planned
Day 14	First dose liposomal amphotericin B; HIV serology negative	Liposomal amphotericin B 3 mg/kg/day commenced	Disseminated histoplasmosis in immunocompetent host confirmed
Day 15	Catastrophic lower GI hemorrhage; hemodynamic collapse; ICU transfer; death	Resuscitation: transfusion, IVF, vasopressors; ICU admission	Fatal outcome: refractory cardiorespiratory failure

## 3 Discussion

The present case illustrates several high‐stakes diagnostic challenges inherent to disseminated histoplasmosis in immunocompetent individuals, particularly in TB‐endemic regions where the ileocecal region serves as a shared anatomical battleground for multiple granulomatous and inflammatory conditions.

### 3.1 Disseminated Histoplasmosis in Immunocompetent Hosts

Progressive disseminated histoplasmosis (PDH) is canonically associated with impaired cellular immunity. However, a subset of cases occurs in immunologically intact individuals, attributed to high‐inoculum exposure, genetic predispositions in innate immune signaling, or environmental factors [6]. Cave exploration—as reported in this patient—represents a well‐documented high‐risk exposure, given the dense accumulation of bat guano that serves as the primary ecological niche for H. capsulatum mycelial growth. Large‐inoculum pulmonary infection from cave aerosols can overwhelm local immune defenses even in immunocompetent individuals, seeding systemic dissemination [7]. This young patient was HIV‐negative with normal cortisol and ACTH levels, which further consolidates the designation of immunocompetence, though a thorough assessment of rare innate immune defects (e.g., STAT3, IL‐12 pathway mutations) was not feasible in the acute clinical setting.

### 3.2 Gastrointestinal and Ileocecal Histoplasmosis

Gastrointestinal involvement in PDH is pathologically frequent, identified in the majority of autopsy series, yet ante‐mortem recognition remains the exception rather than the rule [3]. The ileocecal region is preferentially affected, a predilection shared with both intestinal TB and Crohn's disease, partly attributable to the high density of Peyer's patches in the terminal ileum, which facilitate reticuloendothelial seeding by fungal organisms [4, 8]. Endoscopic appearances—ulcero‐nodular lesions, skip areas, pseudopolyps, and ileocecal valve deformity—are non‐specific and overlap with the colonoscopic spectrum of TB and IBD, as this case exemplifies.

### 3.3 Anchoring Bias and the TB Diagnostic Trap

In high‐TB‐burden countries such as Bangladesh, clinicians commonly operate within cognitive frameworks that favor mycobacterial disease when confronted with ileocecal pathology, weight loss, and lymphadenopathy. Anchoring bias likely contributed to empirical ATT initiation without microbiological confirmation. The failure of ATT after 60 days was a critical signal warranting systematic reconsideration, inclusive of fungal infections [5, 9]. Notably, both GeneXpert and Quantiferon‐TB gold plus were negative, a combination carrying high negative predictive value for active mycobacterial disease.

### 3.4 Diagnostic Mimicry of Crohn's Disease and Elevated Fecal Calprotectin

The radiological and biomarker profile in this case created a compelling—and ultimately misleading—phenotype of Crohn's disease. MR enterography demonstrating multi‐segmental small bowel thickening, elevated fecal calprotectin at 1849.47 μg/g, and fecal inflammatory markers are widely associated with active IBD. However, these findings are not disease‐specific and can be substantially elevated in infectious enteritis, including histoplasmosis, salmonellosis, and intestinal TB [10]. Fecal calprotectin reflects intestinal neutrophilic infiltration irrespective of etiology; its marked elevation should be interpreted as a marker of mucosal inflammation rather than a diagnostic criterion for IBD. The decision to defer immunosuppressive therapy pending histopathological confirmation was clinically appropriate and likely prevented accelerated fungal dissemination.

### 3.5 The Critical Role of Deep Tissue Biopsy and Special Stains

The initial colonoscopic biopsies in this patient yielded only granulation tissue with non‐specific inflammatory infiltration, a pattern documented in prior reports of gastrointestinal histoplasmosis where superficial sampling misses intracellular organisms [11]. The diagnostic breakthrough was achieved through repeat deep tissue biopsy specifically targeting the caecum and ileocecal valve, which yielded lamina propria‐resident histiocytes harboring narrow‐based budding yeast forms with eccentric nuclei—the hallmark morphology of H. capsulatum. The co‐identification of organisms in cervical lymph node aspirate confirmed the disseminated nature of disease. Periodic acid‐Schiff (PAS) and Gomori methenamine silver (GMS) staining are widely advocated to enhance identification of fungal organisms in tissue sections, particularly when organism burden is low [12]. Clinicians and pathologists should maintain a low threshold for requesting fungal stains in cases of unresolved ileocecal pathology, especially following ATT failure.

### 3.6 Mortality, Gastrointestinal Hemorrhage, and Delayed Diagnosis

Gastrointestinal hemorrhage represents one of the most feared and lethal complications of gastrointestinal histoplasmosis. Extensive mucosal ulceration, vascular erosion by the fungal inflammatory process, and consumptive coagulopathy from disseminated infection collectively predispose to catastrophic hemorrhage [13]. In this patient, fatal hemorrhage occurred despite antifungal therapy having been initiated only 72 h earlier, highlighting that treatment may be insufficient to reverse advanced tissue destruction once massive ileocolonic ulceration has developed. Delayed diagnosis—spanning 3 months of symptomatic disease and an additional 14 days of in‐hospital diagnostic workup—likely permitted progressive mucosal destruction beyond the threshold of reversibility. Prior literature has documented mortality rates in disseminated gastrointestinal histoplasmosis ranging from 20% to 50%, with substantially higher rates in cases with diagnostic delays exceeding 4 weeks [14].

### 3.7 Comparison With Published Cases

Case reports and series of gastrointestinal histoplasmosis mimicking Crohn's disease or intestinal TB have been described from North America, India, and South‐East Asia [4, 8, 15]. A consistent theme is diagnostic delay due to initial assumption of mycobacterial or IBD, with the correct diagnosis established through histopathology after therapeutic failure. The present case is distinctive in documenting concurrent cervical lymph node involvement—providing an accessible extra‐gastrointestinal tissue site—and in the fatal outcome due to massive hemorrhage, a complication infrequently detailed in published cases.

## 4 Strengths and Limitation

### 4.1 Strengths

Diagnosis was histopathologically confirmed at two independent sites (cecal/ileocecal valve biopsy and cervical lymph node FNAC), providing high diagnostic certainty. Disseminated disease was documented ante‐mortem. Molecular exclusion of TB via both GeneXpert and Quantiferon‐TB gold plus strengthens the diagnostic conclusion. The case captures the complete diagnostic journey, including ATT failure, misleading biomarker and imaging data, and the value of repeat deep biopsy.

### 4.2 Limitations

Fungal culture and urine/serum Histoplasma antigen testing—which could have accelerated diagnosis—were unavailable. Formal immunological evaluation for innate immune defects was not feasible acutely. The clinical course was truncated by fatal hemorrhage, precluding assessment of antifungal response.

## 5 Conclusion

H. capsulatum, causing disseminated ileocecal disease in immunocompetent individuals, can closely simulate intestinal TB and Crohn's disease across all clinical, endoscopic, radiological, and biomarker parameters. In TB‐endemic settings, failure of empirical ATT must prompt inclusion of fungal etiologies. Deep tissue colonoscopic biopsy with PAS/GMS staining is essential and may require repetition when initial sampling is inconclusive. Empirical immunosuppression for presumed IBD is hazardous without first excluding infection histopathologically. This fatal case illustrates that delayed diagnosis of disseminated histoplasmosis carries a high risk of catastrophic GI hemorrhage, which may be irreversible even after antifungal therapy is initiated.

## Author Contributions

**Rashid Shahriar Sazal:** conceptualization, writing – original draft, methodology, formal analysis, writing – review and editing, project administration, resources. **Sayma Samia Esha:** conceptualization, writing – original draft, formal analysis, data curation, investigation, validation, writing – review and editing. **Abdullah Al Faisal:** supervision, project administration, writing – review and editing, validation, software, formal analysis, funding acquisition. **Md. Abul Kalam Azad:** writing – review and editing, project administration, resources, funding acquisition, investigation, formal analysis, validation. **Khaled Mahbub Murshed:** visualization, supervision, validation, methodology, resources, funding acquisition. **Kazi Ali Aftab:** writing – review and editing, visualization, supervision, funding acquisition, investigation, software, project administration.

## Funding

The authors have nothing to report.

## Consent

As the patient is deceased, written informed consent from the patient's next of kin was obtained according to journal guidelines.

## Conflicts of Interest

The authors declare no conflicts of interest.

## Supporting information


**Figure S1:** Histopathology report of the tissue obtained from the cecal lesion showing granulation tissue with acute and chronic inflammatory cell infiltration. No colonic mucosa was identified in the available specimen, and repeat biopsy was recommended.
**Figure S2:** Cytopathology report of fine‐needle aspiration from the right cervical lymph node showing granulomatous inflammation with numerous intracellular and extracellular organisms consistent with Histoplasma. No malignant cells were identified.

## Data Availability

The data that support the findings of this study are available from the corresponding author upon reasonable request.
