# Supplementary material for: Gastrointestinal Histoplasmosis Mimicking Intestinal Tuberculosis and Crohn's Disease: Ileocecal Involvement in an Immunocompetent Host
Source: Clin Case Rep. 2026 Sep 17;14(9):e73555. doi: 10.1002/ccr3.73555 (PMC13586439; doi:10.1002/ccr3.73555)
Supplement: Supplementary file 1 [file CCR3-14-e73555-s001.pdf]

Histopathology Report

Sample: Tissue from cecal lesion

Gross description:

Specimen received in formalin in a container with name, age, sex, date, ward no, bed no, reg no, hospital name and sample name:

Specimen consists of multiple fragmented grayish white pieces of soft tissue totally measure 0.8X0.6X0.2 cm.

Section code:
A1: Multiple pieces entirely embedded in one block.

Microscopic description/comment:
Sections show granulation tissue only. These reveal infiltration with acute and chronic inflammatory cells

No colonic mucosa is seen in the available material.

Repeat biopsy is recommended.

Supplementary Figure 1: Histopathology report of the tissue obtained from the cecal lesion showing granulation tissue with acute and chronic inflammatory cell infiltration. No colonic mucosa was identified in the available specimen, and repeat biopsy was recommended.

CYTOPATHOLOGY REPORT

Name of Specimen: Cervical lymph node, right

Microscopic Examination:

Smears show lymphocytes, histiocytes and clusters of epithelioid cells in a background of blood. Numerous intracellular and extracellular organism of histoplasma are also seen.

No malignant cell is seen.

Dx: Cervical lymph node, right (FNA): Granulomatous inflammation consistent with histoplasmosis.

Supplementary Figure 2: Cytopathology report of fine-needle aspiration from the right cervical lymph node showing granulomatous inflammation with numerous intracellular and extracellular organisms consistent with Histoplasma. No malignant cells were identified.
